# What We Don't Speak of: Exploring the Impact of Historical Trauma and Discrimination on the Health and Well-Being of Sweden Finns

**DOI:** 10.1007/s11013-026-09969-0

**Published:** 2026-01-19

**Authors:** Mattias Strand, Mona Lindqvist

**Affiliations:** 1 Centre for Psychiatry Research, Department of Clinical Neuroscience, Karolinska Institutet, Stockholm, Sweden; 2 Transcultural Centre, Stockholm Health Care Services, Region Stockholm, Stockholm, Sweden

**Keywords:** Minority health, Labor migration, Diaspora, Cultural competence, Structural competence

## Abstract

Sweden Finns are one of the five officially recognized national minority groups in Sweden. Approximately 3.6 percent of the total Swedish population are either born in Finland or have at least one Finnish-born parent; however, since Swedish national minority legislation explicitly applies a self-identification principle rather than objective criteria in determining who belongs to a national minority group, this figure might not correspond to the number of people who actually view themselves as Sweden Finns. On group level, Sweden Finns have been socioeconomically underprivileged in comparison with the Swedish majority population, and tend to be worse off in terms of somatic and mental health. The aim of this study was to explore the many ways in which official minority status, as well as more subtle and systemic processes of minoritization, affect health and healthcare encounters in the Sweden Finnish population in Stockholm, Sweden. Semi-structured interviews were conducted with 20 adults self-identifying as Sweden Finns. The interview data were analyzed using a thematic analysis framework. In all phases of the study, consultation was sought with a reference group representing the Sweden Finnish community. Three main themes were identified: (1) In-between identities, (2) The meaning of language, and (3) Lack of understanding in Swedish society. The participants underscore the importance of Finnish traditions, food, and customs for their personal well-being. Even so, the findings also point to the many ways in which transgenerational trauma experiences and marginalization contribute to the health challenges faced by Sweden Finns today. The study sheds light on the complex layers of historical trauma and ongoing discrimination and their impact on health and healthcare encounters, underscoring the need for cultural sensitivity within Swedish healthcare and Swedish society.

**Supplementary Information:**

The online version contains supplementary material available at 10.1007/s11013-026-09969-0.

## Introduction

Sweden Finns are recognized as a national minority group in Sweden, alongside Tornedalians, Roma, Jews, and the Sámi (who are also an Indigenous people). The national minority status of the Sweden Finns is based on the longstanding historical roots of Finns and the Finnish language in Sweden (Statens offentliga utredningar, 2017). From the 13th century and up until 1809, the geographical region that is nowadays known as Finland was part of the Swedish nation state. The nature of the relationship between the western and eastern parts of Sweden during these centuries is subject to ongoing scholarly debate (Merivirta et al., 2021); some view it as an example of Swedish colonialism, whereas others argue that such labeling is anachronistic and misleading. During the 16th and 17th centuries, a large population of the so-called Forest Finns migrated from the eastern part of the nation to crown lands in Sweden proper, driven by a combination of scarce farmland and temporary tax exemptions by the Swedish crown. A majority of these internal migrants settled in sparsely populated forest areas in the mid-western region of Sweden proper, where they expanded the arable land by practicing slash-and-burn agriculture on uncultivated terrain. Over the centuries, this historical Finnish-speaking population has gradually integrated into the broader society, leading to the eventual loss of their Finnish language.

During the Winter and Continuation Wars 1939–1944, approximately 70,000 so-called Finnish war children were evacuated to Sweden for humanitarian reasons, and 7100 of them subsequently remained in the country (Mattsson, 2018). In the decades following the Second World War, more than 300,000 Finns came to Sweden as labor migrants. Sweden's policy of official neutrality meant that its industrial sector was largely unaffected by the war, enabling rapid growth in the post-war era (Borg, 2016; Weckström, 2011). To sustain this growth, Sweden needed a steady supply of cheap labor, and this pull effect drew workers from countries such as Finland, Italy, Yugoslavia, and Greece. Finnish labor was particularly attractive due to the geographic proximity. These Finnish workers played a crucial role in building Sweden's booming industry sector, particularly in manufacturing and construction. In contrast, the prospects for young people in war-torn Finland were bleak and the opportunity to migrate to Sweden for work was usually seen as exciting (Liimatainen, 2022a; Lukkarinen Kvist, 2006). The Finnish post-war labor migrants were mostly young, single, and relatively low educated. Finns in Sweden were the subject of much prejudice and they and their children suffered far-reaching discrimination (Ågren, 2006; Beckman, 2018; Kuosmanen, 2001; Liimatainen, 2022a; Lukkarinen Kvist, 2006; Weckström, 2011). Sweden Finns have described life in Sweden during the 1960s and 1970s as characterized by a strong sense of in-group community, but there is also a darker history of marginalization and many felt compelled to hide their Finnish background in various ways, including not passing on the Finnish language to their children (Weckström, 2011).

During the 1970s and 1980s, the Finnish economy bloomed. Some labor migrants chose to return to Finland, but many ultimately remained in Sweden (Lukkarinen Kvist, 2006). From the 1980s and onwards, Finns have continued to move to Sweden for work or study; this generation of migrants tends to be more highly educated, reflecting the rise of Finland as an economically and technically highly advanced country (Lukkarinen Kvist, 2006).

The number of individuals in Sweden who are either born in Finland or who have at least one Finnish-born parent was 383,000 in 2024 (Statistics Sweden, 2025), i.e., approximately 3.6 percent of the total Swedish population. Up until 2016, people born in Finland were the largest immigrant group in Sweden (after which Syrian migrants have been more numerous). However, Swedish national minority legislation explicitly makes use of a self-identification principle rather than any objective criteria in deciding who belongs to a national minority group, and having a Finnish background is therefore not necessarily the same as identifying as a Sweden Finn. Formally, the term Sweden Finns refers to Finnish-speaking residents in Sweden and their offspring (who may or may not speak Finnish), but the emphasis on self-identification—including the freedom of any individual to view themselves as *not* belonging to a national minority group, regardless of mother tongue and origin—contributes to an inherent conceptual fluidity.

In Sweden, national minority status is determined on the basis of several criteria: (a) the group must have a distinct sense of cohesion and occupy a non-dominant position in society relative to the rest of the population; (b) the group must display a religious, linguistic, traditional, and/or cultural distinctiveness not shared with others; (c) it must have a clear desire and aspiration to preserve its identity; and (d) there must be historical and longstanding ties to Sweden. In 2000, Sweden acceded to the Council of Europe’s Framework Convention for the Protection of National Minorities and the European Charter for Regional or Minority Languages. In connection with this, national minority policy was established as a distinct policy area, which was subsequently reformed in 2009 and 2019. The overarching objective of this policy is to protect the national minorities and to strengthen their opportunities for participation and influence, as well as to support the five historical minority languages—Finnish, Meänkieli, Romani chib, Yiddish, and Sámi—in order to ensure their continued vitality. Swedish national minority stipulates that the minority languages are to be protected and promoted, that the national minorities are to be able to preserve and develop their culture, and that public authorities are obliged to inform about the minorities’ rights and to ensure their participation in matters affecting them. Local authorities (i.e., municipalities and regions) are also mandated to set goals and guidelines for their minority-policy work. Arguably, the national minority legislation has contributed to an increased recognition and visibility (Liimatainen, 2022a), as well as to stronger policy structures and monitoring. Even so, implementation at municipal and regional level remains uneven, and a recent official report concluded that the government’s efforts are insufficient to achieve the objective of keeping national minority languages alive, citing coordination failures, shortages of teachers in minority languages, and short-term rather than long-term planning (Swedish National Audit Office, 2025).

Many of the rights associated with national minority status concern the minority languages; for instance, Sweden Finns have the right to use the Finnish language in communication with governmental and municipal authorities (Statens offentliga utredningar, 2020). Historically, however, they have had to struggle for their right to actively use their languages (Lainio, 2014) and, as touched upon above, parents have often felt a pressure to abstain from teaching children their minority language out of fear of stigmatization (Rasmussen & Nolan, 2011; Weckström, 2011). Thus, it is fully possible to self-identify as a Sweden Finn without mastering the Finnish language—this is not uncommon among second- and third-generation Sweden Finns (Statens offentliga utredningar, 2017). People in Sweden with a Finnish background may of course also view themselves as Swedes, Finns, and Sweden Finns all at once. Due to the self-identification principle, it is possible to identify as belonging to several national minorities—e.g., Sweden Finn *and* Sámi—or as Sweden Finn *and* African Swedish, for instance, if one’s parents have migrated to Sweden from different countries.

Like other labor migrant populations, Sweden Finns as a group have been socioeconomically underprivileged in comparison with the Swedish majority population (Liimatainen, 2022a; Lundström, 1996). This tends to have a negative impact on health. It is by now well established that individuals with a Finnish background in Sweden tend to be worse off in terms of both somatic and mental health compared to the rest of population. For instance, Finnish-born women and men in Sweden, as well as women and men born in Sweden with at least one Finnish-born parent, display substantially elevated total mortality rates compared to the Swedish-born population and their offspring (Manhica et al., 2015; Östergren et al., 2023). Considering that most other migrant groups to Sweden display *lower* total mortality rates compared to the Swedish-born population (Wallace, 2022), these elevated rates are particularly notable. In terms of mental health, individuals with a Finnish background in Sweden are more often diagnosed with various serious psychiatric disorders, such as schizophrenia and other psychotic disorders (Saraiva Leão, 2006). Alcohol and other substance use disorders are also more common (Folkhälsomyndigheten, 2019; Hjern & Allebeck, 2004; Östergren et al., 2021, 2023; Saraiva Leão, 2006). Moreover, alarmingly high suicide rates have consistently been reported among the Finnish-born population in Sweden (Hjern & Allebeck, 2002; Johansson et al., 1997; Mäkinen & Wasserman, 2003; Westman, 2006), although the most recent study on suicide in this group was published almost 20 years ago, indicating an obvious research gap. It is also worth noting that some studies including comparisons with Finns in Finland suggest that individuals with a Finnish background in Sweden occupy an intermediate position in terms of health. For example, alcohol consumption and alcohol-related mortality in this group are lower than among Finns in Finland, but higher than among the Swedish majority population (Östergren et al., 2021; Saarela & Kolk, 2020). Given these patterns of substantially worse health among individuals with a Finnish background in Sweden, it is somewhat surprising that the health and healthcare experiences within this population have not been more thoroughly explored through qualitative studies. More qualitatively oriented research is needed to further explore context and causal pathways behind the observed patterns and to guide potential preventive measures.

## Aims

The present study was initiated following requests from the regional Sweden Finnish community to Region Stockholm, the public body responsible for healthcare in Stockholm County, which subsequently funded an investigation into the health of Sweden Finns in the region. The overarching aim of the study is to explore the many ways in which official minority status, as well as more subtle and systemic processes of minoritization, affect health and healthcare encounters among the Sweden Finnish population in Metropolitan Stockholm, Sweden. In the government inquiry leading up to the extension of the Swedish national minority legislation in 2019, it is explicitly noted that a knowledge gap exists concerning the health of the national minorities and that minority-specific cultural and linguistic competence in healthcare is urgently needed (Statens offentliga utredningar, 2017). With this study, we hope to contribute to bridging this gap by explicitly focusing on discourses of health and illness in the minority identity context—discourses which, for Sweden Finns, involve legacies of historical trauma and migration that are not always fully acknowledged in majority-Swedish society.

## Methods

### Study Design

In this qualitative study, lived experiences of health and healthcare encounters shaped by minority status among adult Sweden Finns in Stockholm, Sweden were explored through individual in-depth interviews. In an effort to involve stakeholders throughout the research project (Thambinathan & Kinsella, 2021), a reference group of four individuals was established by inviting organizations representing the Sweden Finnish community in Stockholm to appoint their own representatives. The reference group contributed collaboratively in identifying relevant areas of interest, designing the interview guide, outreach and recruitment, and in continuous follow-up of the study procedures and findings.

A semi-structured interview guide was created based on available previous research and input from the Sweden Finnish reference group. Some of the questions were also inspired by relevant sections of the Cultural Formulation Interview, included in the fifth edition of the *Diagnostic and Statistical Manual of Mental Disorders* (American Psychiatric Association, 2013). The final interview guide covered topics such as Sweden Finnish self-identification, the significance of cultural identity for health and illness, risk factors and resilience, experiences in healthcare and with other relevant government authorities, experiences of discrimination, and needs and expectations for future healthcare encounters. A translated version of the interview guide is provided as supplementary material.

### Participant Recruitment

Recruitment was mainly organized through the Sweden Finnish community organizations represented in the project reference group (Sverigefinska Riksförbundet/Ruotsinsuomalaisten Keskusliitto and Sverigefinska Ungdomsförbundet/Ruotsinsuomalaisten Nuorten Liitto). Further recruitment efforts included the network of the Transcultural Center in Region Stockholm, the Finland Institute in Stockholm, the Section for Finnish at Stockholm University, Sweden Finnish schools in the region, and the national minority website Minoritet.se. Snowball recruitment, by which research participants were encouraged to invite Sweden Finnish relatives and friends to participate, was also used.

Inclusion criteria for participation in the study were (a) age ≥18 years, (b) self-identification as Sweden Finn, and (c) being able to provide informed consent. No explicit exclusion criteria were employed, as long as the inclusion criteria were satisfied. Recruitment ads were developed in collaboration with the Sweden Finnish reference group to ensure that self-identification was explicitly highlighted as a guiding principle.

### Interview Procedure and Analysis

Individual semi-structured interviews lasting between 50 and 100 minutes were conducted with all research participants. All interviews were conducted in 2023. Participants were offered to speak Swedish or Finnish (or both) during interviews. ML conducted eleven interviews and MS conducted nine interviews. Audio recordings from the interviews were transcribed verbatim and anonymized by altering potentially identifiable content such as name, place of birth, and occupation. Participant quotes were then translated from Finnish to Swedish by ML, and at a later stage to English. Translation was complicated at times, since it is not always possible to find linguistically and culturally equivalent terms between the languages, and rather than striving for a literal translation, contextual meaningfulness was prioritized (van Nes et al., 2010).

The interview data were analyzed using a thematic analysis framework (Braun & Clarke, 2021). As a first step, both researchers independently read all transcribed interviews multiple times and preliminarily identified potentially relevant themes and subthemes. These analyses were then discussed jointly and refined in an iterative process, involving alternating bottom-up (i.e., data-grounded) and top-down (i.e., assessing if preliminary themes and subthemes ‘worked’ by reapplying them on the data) perspectives. In this phase, data mapping—by which the thematic analysis was visualized in multiple ways, including color-coded charts, spider diagrams, and conceptual maps, to explore overlaps, tensions, and hierarchical relations among themes—was a helpful tool for deepening our understanding of the interview data. This process allowed us to refine theme boundaries, confirm analytical consistency, and consider alternative interpretations before finalizing the thematic structure. When consensus was reached on final themes and subthemes, we reassessed the interview data accordingly and illustrative quotes were chosen.

### Ethics and Preregistration

The research presented here is approved by the Swedish Ethical Review Authority (no. 2022-05163-01). Verbal and written informed consent were obtained from all participants. The study protocol has been preregistered on the Open Science Framework (osf.io/45jnu).

### Positionality

The research team consisted of two clinician-researchers working at the Transcultural Center in Region Stockholm. MS is a psychiatrist and associate professor specializing in transcultural psychiatry and holds a PhD in Medical Science. His work is situated within the Swedish higher education system and the field of psychiatry, both of which have historically centered majority perspectives on ethnicity. He identifies as male, cisgender, and White—although in a Swedish, majority-White context, this seldom constitutes a marked or meaningful social position. He was born in Sweden and although his family has substantial Finnish heritage (as do many Swedes), he did not grow up speaking Finnish and has never self-identified as Sweden Finn. His connection to the Sweden Finnish community is therefore primarily historical and academic rather than personal, which may both enable analytical distance and limit emic understanding. He lacks personal experience of linguistic marginalization, which may constrain his insight into language-based exclusion experienced by minority communities. While he benefits from majority privilege, his ancestral connection to the Forest Finn population provides a tenuous link to the history of Sweden’s national minority groups, offering both identification and distance in relation to questions of ethnicity.

ML is a licensed clinical psychologist and psychotherapist, and holds a PhD in Sociology. She was born in Finland and moved to Sweden as an adult. She identifies as a Sweden Finn with a bilingual background and is fluent in both Finnish and Swedish. This bilingual position provides an insider perspective that informs her understanding of cultural belonging, displacement, and hybridity. Drawing on an emic approach, she seeks to grasp meanings and experiences from within through the participants’ own concepts and understandings of culture, language, and emotional worlds. Language, in this context, is not merely a means of communication but a carrier of culture. Mastery of Finnish enables access to layers of meaning that are embedded in linguistic expressions, metaphors as well as silences—elements that often resist translation. To speak and think in Finnish is to inhabit a particular cultural rhythm and emotional landscape; to move between Finnish and Swedish is to navigate between worlds. This bilingual movement shapes her sensibility as both observer and participant, allowing for a nuanced understanding of how identity and belonging are negotiated in translocal and translingual spaces.

As researchers and authors, we were thus able to apply both insider and outsider perspectives on the data. Importantly, we offered participants the option of conducting the interview in Finnish or Swedish (or both), although most individuals who self-identify as Sweden Finns has a thorough knowledge of the Swedish language. This choice was based not only on the linguistic rights associated with the recognized national minority status, but on the fact—as outlined above—that human experience is intimately shaped by the context of language (Abfalter et al., 2021; Caretta, 2014) and that it might be easier to express experiences of health and healthcare in the language that is closest to one’s heart.

## Results

A total of 20 research participants—17 women and three men—were recruited. Their mean age was 53 years (median: 61.5 years, range: 25–78 years). Fifteen participants were born in Finland; 13 of them moved to Sweden as adults, whereas two migrated as children with their parents. Three participants were born in Sweden with at least one Finnish-born parent and identify as belonging to the ‘second generation.’ Last, two participants consider themselves as belonging to a ‘third generation,’ i.e., one or more of their grandparents migrated to Sweden from Finland. One participant had secondary-level education (e.g., highschool), all others had at least some post-secondary education.

Eight out of 20 participants chose to conduct the interview primarily in Finnish. All of these participants were women with a post-secondary education, they had migrated from Finland to Sweden as young adults, and they all considered Finnish their first language. Still, it is important to recognize that all participants who chose to speak Finnish during the interview also spoke Swedish fluently, although they preferred to use the Finnish language when discussing experiences related to health and illness.

Three main themes were identified, all of which involve several subthemes of importance for the participants’ experiences of health and healthcare encounters shaped by minority status:

1. In-between identities
2. The meaning of language
3. Lack of understanding in Swedish society

These themes and subthemes are described in more detail below.

### In-Between Identities

This theme revolves around a substantial element of mixedness in terms of personal identity among the participants; a state of being in-between Swedish, Finnish, and Sweden Finnish identities, norms, and traditions that relates to experiences of health and well-being in numerous ways. Three subthemes can be identified: Mixedness and ambivalence about the very concept of Sweden Finns, thinking about and relating to pervasive stereotypes about the group, and the importance of maintaining traditions.

#### Ambivalence About the Concept of Sweden Finns

Several participants describe how they see themselves as both Swedish and Finnish—this is particularly common among those who are born in Finland and subsequently moved to Sweden during childhood and adolescence or as adults. For most participants, this mixedness is not described as problematic but as a simple fact of life. However, some participants emphasize either their Swedish or Finnish identity (most often the latter of the two) over any notion of mixedness or being in-between:

Just because we look the same, many people believe that we are the same, but we’re really not. […] I’ve lived in Sweden for a long time and I understand the Swedish way of being, but it’s not my way. I live a Finnish life in Sweden. (Participant 19; woman in her 70s born in Finland)

Several participants describe other elements than Swedishness and Finnishness as vital parts of their own ethnic and cultural identity. For example, one of their parents might be born in a third country or they view their Sámi or Tornedalian roots as equally important*.* An idea that several participants raise is that a stereotypical Swedish notion of supposedly Finnish traits actually more closely describes habits and mindsets shaped by life in the rural north of either country:

If you move from a town in northern Finland to a town in northern Sweden, the contrast is not as big as if you migrate from northern Sweden to Stockholm or Helsinki [i.e., the capitals of Sweden and Finland, respectively, both situated in the south], or from northern Finland to Stockholm or Helsinki. That’s a huge difference. There’s a certain shared experience of the rugged life in the Circumpolar North that’s not present in the boulevards of Stockholm. (Participant 12; woman in her 60s born in Finland)

A smaller number of participants embrace an explicitly cosmopolitan or transnational identity, in which Sweden and Finland as individual nations are of less importance:

I’m slightly complicated. […] I say that I’m a European with Finnish background and right now I live in Sweden. It’s a fluid identity. (Participant 3; woman in her 70s born in Finland)

Whereas identifying as both Swedish and Finnish is common, most participants express an ambivalent relationship to the officially recognized terminology—i.e., Sweden Finns—used in national minority politics and legislation:

I don’t use that term [i.e., Sweden Finn]. If someone asks me where I’m from, I tell them I’m from Finland. It’s easier, for some reason. I can’t explain it. I simply never learned to use [Sweden Finn]. (Participant 8; woman in her 60s born in Finland)

Not least, those participants who belong to the ‘third generation,’ i.e., individuals with one or more grandparents born in Finland, might feel like they are too far removed from what they see as ‘real’ Finnishness to call themselves Sweden Finns:

[I don’t use it] because I don’t think I’m Finnish enough to be able to claim it. I know it sounds stupid, but that’s how I feel. (Participant 1; woman in her 30s with grandparents born in Finland)

While some participants do not outright reject the concept of Sweden Finns, they express discomfort with the term Finn specifically, given its historical use in Sweden which carries not only derogatory but also racially charged undertones.

#### Stereotypes: Negative, Positive, and a Little Bit of Both

Many participants discuss how being Finnish or Sweden Finnish have tended to be associated with various negative cultural stereotypes and tropes, which are still encountered in Swedish popular culture and mass media as well as at work and in private relationships:

What I hear a lot is that we’re all alcoholics and we all carry a knife. (Participant 10; woman in her 20s with parents born in Finland)

Some participants describe that whereas these negative stereotypes certainly remain, they might have been more prevalent and crude 30 or 40 years ago:

[My grandpa] told me about stuff similar to what my racialized friends talk about today. For example, if a crime had been committed and he read about it in the newspaper, he thought to himself: “God, I hope it’s not a Finnish person because then we’ll all be blamed as a group.” (Participant 1; woman in her 30s with grandparents born in Finland)

Even so, it is evident that many negative stereotypes about people with a Finnish background still prevail in Swedish society. Several participants describe how they might, for example, choose to participate in jokes or not make a big deal out of them as an attempt to handle the stereotypes they encounter:

It can feel as if you ruin the mood if you say: “C’mon guys, I feel offended.” Those few times that I’ve tried to say something about it, they’ve just responded: “What, we thought Finnish people were supposed to be tough?” It’s hard to deal with it in a good way. (Participant 11; man in his 30s with parents born in Finland)

Importantly—and this will be touched upon again in the section about cultural and structural competence in healthcare below—participants also acknowledge excessive alcohol use as a real problem in some parts of the Sweden Finn population, while simultaneously expressing a will to nuance and contextualize this fact.

In parallel to the various negative stereotypes, participants also mention a number of somewhat more positive stereotypes commonly associated with Sweden Finns. Not seldom, these also relate to the labor migration history of the group, but from a perspective that emphasizes diligence and honesty (although they can, of course, still be viewed as representing a distorted, majority-population view on Sweden Finns):

Give me a task and I’ll make sure to do it. It’s an inherited trait, that’s how it was at home. You don’t leave things undone. Clean out your own house before you start talking about other’s. (Participant 12; woman in her 60s born in Finland)

Nevertheless, it is obvious that even those stereotypes that come in a more advantageous form are rarely completely positive:

Sisu [i.e., a Finnish expression encompassing grit and resilience, often regarded as a semi-official Finnish national character] or endurance can be positive and negative. You don’t easily give up, but it can also mean that you don’t let go of something even if it ends up killing you. (Participant 15; woman in her 60s born in Finland)

Consequently, some participants view the very notion of ‘positive’ stereotypes as deceptive and ultimately harmful.

#### The Importance of Traditions for Health and Well-Being

Finally, a recurrent element of the mixed identities described by the participants is the special emphasis put on Finnish customs and traditions in (re-)creating and upholding Finnishness in Sweden. Food and cooking in particular act as important links to a (supposedly) Finnish heritage:

We’ve always had Finnish Christmas food and it matters a lot to me. This Christmas was actually the first I spent separated from my family […] but it still felt important to make Karelian pasties and carrot casserole and all that. (Participant 1; woman in her 30s with grandparents born in Finland)

Another highly important Finnish ritual (sometimes closely associated with certain foods) is the habit of sauna bathing:

Sauna, sauna, how can you live without a sauna? The sauna is my temple, my church. (Participant 18; woman in her 60s born in Finland)

A wood-fired sauna with birch twigs is good, because it makes it easier to breathe for an asthmatic like me. I relax and feel good, as simple as that. (Participant 6; woman in her 50s with parents born in Finland)

Other important cultural aspects of Sweden Finnishness mentioned in the interviews are music, dance, handicraft, and a particular sense of humor. Common to these narratives is a sense of impending loss. Since the Finnish part of one’s identity cannot always be taken for granted (or ‘claimed,’ as one participant describes it), Finnish traditions must be upheld against a hegemonic and arguably more anemic Swedish majority culture that appears to possess a very limited knowledge of its neighboring country.

### The Meaning of Language

Language (and lack of language) is, of course, closely tied to cultural identity and heritage. Even so, the meaning of language stands out in the interviews as an important theme in its own right. Three subthemes can be identified: Embracing differences between Finnish and Swedish, missing a language that has been lost due to stigma and structural discrimination, and language as an object of ridicule.

#### Embracing Differences Between Finnish and Swedish

Several participants describe how the Finnish and Swedish languages are so fundamentally different in terms of vocabulary, idioms, and emotional content that some things are not easily expressed in Swedish. Importantly, language differences—as well as cultural differences in preferred communication styles in a broader sense—can contribute to misunderstanding in healthcare and other settings. For instance, several participants experience a typically Swedish style of interaction as more indirect and harmony-seeking compared to how they use the Finnish language:

It’s so much easier when healthcare providers speak Finnish or when they are familiar with how Finnish people express themselves. You can get straight to the point and don’t need to explain things in a certain way. (Participant 13; woman in her 20s born in Finland)

#### Missing a Language Lost

Although many participants view the Finnish language as highly expressive, there are also barriers to using it. An important obstacle is the historical resistance to bilingualism in Swedish society, based on ideas about child language development that are now seen as obsolete. This affects the children and grandchildren of Finnish immigrants in particular. Moreover, many Finnish-speaking parents chose not to teach their children Finnish for fear of societal stigma and gave them Swedish-sounding names in order to protect them from harassment. Often, stigmatization and official legislation have co-contributed in raising barriers to adequate support for Sweden Finns in learning the Finnish language. In contrast to some of the other national minority languages, Finnish is not an endangered or vulnerable language. Still, for the Sweden Finnish community, limited Finnish proficiency among younger generations results in loss of access to cultural heritage as well as in barriers to interaction with exclusively Finnish-speaking relatives. Another issue raised by several participants is the tendency among Finnish-born immigrants to gradually lose the Swedish they have acquired as adults as they grow older, due to cognitive impairments:

Your Swedish language-skills are not firmly rooted, because you learned the language at work [as an adult labor migrant] and then you lose it. Perhaps [healthcare providers] need to consider that when it comes to elderly Finns. (Participant 3; woman in her 70s born in Finland)

#### Being Ridiculed and Subjected to Microaggressions

Another important barrier to using the Finnish language is related to experiences of being ridiculed by others, closely associated with the perception of Finnish as a minority and immigrant language:

It’s sooo tiresome! There’s always that person that needs to show that they “know” Finnish and starts saying all the curses they know or counting “one, two, three”. Imagine being in Finland and walking up to somebody from Sweden and going: “One, two, three, pussy, dick, hell”. It’s just weird. (Participant 14; woman in her 20s born in Finland)

### Lack of Understanding in Swedish Society

This overarching theme includes descriptions of how a limited understanding among healthcare providers and others in Swedish society with regard to the history of Sweden Finns and to the particular social and cultural determinants of health that affect the Sweden Finnish population leads to miscommunication and suboptimal treatment. Three subthemes can be identified: Cultural differences in how distress is expressed and communicated, a lack of acknowledgement of the pervasive impact of war on the Sweden Finnish community, and the implications of the history of scientific racism in Sweden targeting Finns and Sweden Finns.

#### Expressing Distress

This subtheme builds on what could also be seen as a stereotype about Sweden Finns in general and Sweden Finnish men in particular, namely the notion that they tend to endure in their illness, make light of symptoms of somatic and mental distress, and only reluctantly seek help from healthcare providers and other potential sources of support. Several participants describe this tendency as real and as a cultural characteristic of Sweden Finns as a group:

Sometimes I’m caught behaving like that myself. For example, when I had symptoms of cardiac arrhythmia. I was thinking that I didn’t need to get it checked, but my partner made me book an appointment. That’s just one example. I usually think: Why go to the emergency department and add to their burden when I know how terribly busy they are? I feel pretty ok, why bother them? (Participant 9; man in his 60s born in Finland)

For some, this tendency also builds on experiences of being misunderstood, neglected, or even discriminated against when they have in fact tried to access adequate help:

I always try to be matter-of-fact when it comes to my medical symptoms, I try to describe them. […] I try to concretize and not bring my feelings into it. Perhaps that gives them the impression that I’m not that bothered about it, I don’t know. (Participant 11; man in his 30s with parents born in Finland)

A widespread prejudiced notion of Sweden Finns as taciturn, tough, and stoic might contribute to overly optimistic assessments and tactlessness in healthcare settings. This involves ideas about the semi-official Finnish national character of sisu (touched upon above) as a resource *and* an obstacle to receiving support:

For instance, when my dad was dying in a hospital in 2001, one of the nurses told him: “Just get up, there’s nothing wrong with you, you’re Finnish.” Luckily, my brother was there and told them off. (Participant 6; woman in her 50s with parents born in Finland)

Many participants, however, take another perspective on the supposed reluctance of expressing distress and illness among Sweden Finns. They acknowledge that their idioms of distress and ways of communicating around help-seeking might seem subdued and avoidant in a majority-Swedish context, but they also underscore that whenever they have the opportunity to see Finnish or Sweden Finnish healthcare professionals, their symptoms are readily understood and acted upon:

I lived in [a town close to the Finnish border] when I was in college and I got to see the same family doctor for a long time. She learned how to read my facial expression, because she knew that I wasn’t exactly going to tell her that I was in pain. (Participant 10; woman in her 20s with parents born in Finland)

Descriptions like these imply that when Finnish or Sweden Finnish healthcare professionals are faced with the very same patient presentations and symptoms as their majority-Swedish colleagues, they somehow possess the cultural sensitivity that is needed to ‘read’ them in a meaningful manner. This includes emic knowledge about and insight into linguistic and cultural aspects as well as structural issues that shape the community:

It’s easier to explain the issues that I have with my father or the family problems we experience to someone that has some insight into the family dynamics of Sweden Finnish families, how they usually function. (Participant 11; man in his 30s with parents born in Finland)

#### The Impact of War

A recurrent topic in the participant narratives is the pervasive impact of wars on Finnish society and, consequently, on the Sweden Finnish community. A number of devastating wars were waged on Finnish ground in the 20th century: The Finnish Civil War of 1918 as part of the transition to independence from Russian rule, the Kinship Wars of 1918–1922, the Winter War of 1939–1940, the Continuation War of 1941–1944, and the Lapland War of 1944–1945. Many participants explicitly link these experiences of war and war trauma to some of the health issues that continue to affect the Sweden Finnish population, not least those associated with excessive alcohol use:

There’s hardly one family in Finland that didn’t lose someone during the wars. Surely that’s had a huge impact. All the violence and the alcohol. […] My guess is that’s the single most important reason. (Participant 9; man in his 60s born in Finland)

How they communicated their wartime experiences? It was a total silence. Either you didn’t speak about your experiences with anyone or you drank with other former soldiers and repeated the same stories over and over again. (Participant 20; woman in her 70s born in Finland)

Importantly, the horrors of war did not only affect the soldiers in active combat; the participants also discuss and acknowledge the widespread impact of war and post-war trauma on a collective and transgenerational level:

The war didn’t only cause poverty. Everything was broken, destroyed. Houses, people, relationships, everything. (Participant 17; woman in her 60s born in Finland)

We have some sort of melancholy ingrained in our souls. I recognize it when I talk to my Finnish friends. It’s been with us since we were children. (Participant 8; woman in her 60s born in Finland)

Some participants emphasize the contrasts between Sweden and Finland in the post-war era. Whereas Sweden was largely unharmed by the Second World War, war-torn Finland was plagued by destruction, poverty, and unemployment, and widespread industrialization and modernization occurred much later in Finland. This was a strong driving force behind the labor migration to Sweden in the post-war years (alongside the pull effect of the booming Swedish industrial sector) and has affected several first-generation migrant participants:

I experienced a time when electricity didn’t exist. In the winter, our well froze and we had to melt ice for the animals to drink. […] I hadn’t seen a water tap before we moved to Sweden. You might say I come from the 19th century. (Participant 12; woman in her 60s born in Finland)

Although the tremendous impact of war is emphasized in almost all of our informant narratives, many also highlight how most Swedes appear to know very little about the historic hardships endured by their neighboring country. One informant mentions that a younger generation of Swedes simply has not learned about the harsh reality of war; for example, when the Winter War is mentioned in Swedish elementary school education, a main focus tends to be on the participation of the Swedish Volunteer Corps, depicted in a vaguely romantic light. In effect, the wars are forgotten but the suffering remains in the form of prevailing negative stereotypes depicting Finns and Sweden Finns as melancholic, violent, and plagued by alcoholism.

#### The Heritage of Scientific Racism

Yet another aspect of the lack of knowledge and awareness in Swedish society with regard to the health and well-being of Sweden Finns has to do with the heritage of scientific racism. In early 20th century, views of Finns as racially different and inferior dominated in Swedish academic circles and society at large. Although the language used subsequently shifted, the general idea of Finns and Sweden Finns as a subordinate Other remained:

Just because the structural and social framework has changed, stereotypes can still linger on. The view of Finns as a foreign race was present in Swedish school books up until the end of the 1960s. Then you remove the word “race” and replace it with “culture”, “ethnicity”, or “nationality”, but the description remained the same well into the 90s. (Participant 2; man in his 30s with grandparents born in Finland)

A couple of participants specifically mention what they see as a prevailing historical trauma involving the celebrated Swedish researchers Anders and Gustaf Retzius, who accumulated a large collection of Finnish skulls that they used for phrenological pseudoscientific purposes. Some of these skulls were dug up and stolen from graveyards during an expedition to Finland in 1873, and they have been kept at Karolinska Institutet in Stockholm ever since (where they remained at the time of our participant interviews). In September 2024, the skulls were finally repatriated to Finland.

## Discussion

Sweden Finns have a profound history of migration, sociocultural integration, and identity formation in Sweden. The Sweden Finnish population has, over generations, contributed to the cultural and demographic fabric of the nation. Despite their longstanding presence, Sweden Finns have encountered continuous discrimination and cultural marginalization, impacting both their linguistic and cultural heritage and their overall sense of belonging. The findings from the present study point to the many ways in which transgenerational trauma, marginalization, and discrimination within Swedish society might have contributed to the health challenges faced by Sweden Finns today, highlighting how these factors may give rise to a higher prevalence of psychological distress, substance use, and self-harm (Weckström, 2011). In considering the historical trauma associated with Finland’s wartime past, our study sheds light on the complex layers of past and present stereotyping and discrimination and their impact on health and healthcare encounters, underscoring the need for cultural sensitivity, recognition, and respect within Swedish healthcare and Swedish society.

### The Othering of Finns in Sweden

In general, our research participants describe a fairly uncomplicated relationship to national and ethnic terms such as ‘Swedish’ and ‘Finnish.’ In some situations, they tend to experience themselves as mostly Finnish, whereas in other contexts, their Swedishness is more firmly emphasized; often, both terms are central to their experience of personal and cultural identities. However, and perhaps somewhat surprisingly, the specific minority-legislative terminology of ‘Sweden Finns’ is usually seen as less relevant. Those few participants who have explicitly embraced the concept of Sweden Finnishness are typically more involved in a political minority discourse. They tend to be either slightly older—having lived through and participated in the early days of struggle for national minority rights in Sweden in the 1970s and 1980s—or to belong to a third generation that has discovered and claimed their Sweden Finnish identity as adults. In a sense, this mirrors findings from studies of other migrant groups, for whom ethnic identity and associated ideas of origin and authenticity become more important for the children and grandchildren of immigrants to a new country in an ongoing negotiation of social identities (Nahachewsky, 2002). Importantly, for healthcare services that wish to reach the Sweden Finnish community in attempts to address ethnic disparities in health, it might actually be useful to go beyond the officially recognized minority terminology and explicitly target a wider group of individuals with a Finnish background.

Regardless of terminology, nearly all participants underscore the importance of Finnish traditions, food, and customs—the Finnish sauna culture, not least—for their personal well-being. For some, there are direct health aspects, such as the positive impact of sauna bathing on the respiratory system (Kunutsor et al., 2017). More often, however, maintaining Finnish traditions is tied to a more general sense of community and belonging within the group, whether or not one embraces the ‘Sweden Finn’ label. Moreover, there is often an implicit sense of threat and impending loss associated with the emphasis of Finnish culture heritage, made more urgent by the ‘exile’ in Sweden. This notion is further underscored by the fact that (White) individuals with a Finnish background can typically ‘pass’ as (White) Swedes, especially so if they have grown up in Sweden and do not speak with an accent (Liimatainen, 2022a). This contributes to turning Finnishness in Sweden into something almost invisible from a majority-population point of view, which, in turn, might make it more difficult to identify and address discrimination and ethnic disparities.

Several participants talk about how they position themselves in relation to a heritage of scientific racism in Sweden, with the issue of skull repatriation briefly described above serving as a striking illustration. In early 20th century, leading racial scientists Herman Lundborg, Frans Josua Linders, and Rolf Nordenstreng described Finns as belonging to a so-called East Baltic race, which was seen as Caucasian but nevertheless ‘Mongolized’ and, thereby, inferior to the Nordic race of the majority-Swedish population (Kemiläinen, 1998). Whereas Swedes were characterized as individualist, entrepreneurial, and scientifically minded, Finns were described as collectivist, introvert, and brooding. After the Second World War, scientific racism was generally denounced and Finns were incorporated into the fluid and porous notion of ‘mainstream’ Whiteness (Beckman, 2018; Liimatainen, 2022b), similar to how groups such as Italians, Eastern Europeans, and Ashkenazi Jews have gradually come to be regarded as White. Even so, racially charged stereotypes of Finns and Sweden Finns as subordinated Others have lived on in Sweden (Beckman, 2018). This notion has probably been reinforced by the fact that the children of Finnish labor migrants to Sweden have often grown up in urban neighborhoods characterized by a working-class, hyperdiverse migrant population where Finnish has been one of many foreign languages spoken among children and teenagers (Weckström, 2011). Thereby, a nexus of Sweden Finnishness, working-class identity, and urban youth cultural hybridity has been established, resembling what is sometimes referred to as *new ethnicities* in an international context (Back, 1996; Hall et al., 1996).

Experiences of being seen as merely ‘almost White,’ similar to those of our participants, have been described among first- and second-generation Polish and Russian migrants to Sweden (Lönn, 2018; Runfors, 2021). The concept of *racialized temporality* (Solomon, 2019), by which various migrant Others are seen as somewhat less modern and progressive—as if inhabiting a different time zone, if you will—is relevant to this discussion. Similar to how Russians in Sweden describe the attitudes they face (Lönn, 2018), Finns have often been depicted in Swedish popular culture as remnants of the 1970s and 1980s; the stereotypical Finnish man on Swedish television shows sporting a tacky moustache and a blonde mullet (a look that has, arguably, been rehabilitated by the neo-hipster movement of recent decades). Several of our participants describe how it is still common, and more or less acceptable, to make jokes about Finns in Sweden in a way that one would no longer joke about most other migrant groups. This particular experience mirrors that of many Asian Swedes; another (highly diverse) population that are still regularly being made fun of in a racist and stereotypical manner in Swedish films and television (Hübinette, 2024).

In our interviews, there is a certain ambivalence concerning the prevailing health behavior stereotypes experienced by many Sweden Finns. For instance, it can be difficult to navigate tiresome sociocultural tropes of Sweden Finns as prone to alcohol abuse. Several participants make a point of the fact that they personally drink very little alcohol; even so, alcohol and other substance use disorders have undoubtedly been more common among individuals with a Finnish background in Sweden (Folkhälsomyndigheten, 2019; Hjern & Allebeck, 2004; Östergren et al., 2021, 2023; Saraiva Leão, 2006). Notably, minoritized groups often tend to be blamed for their own misfortune (Brewis & Wutich, 2019; Greenhalgh & Carney, 2014), which may lead to a vicious circle by which younger generations grow up incorporating a self-image of addiction and violence as an inherent trait of the population rather than something that has been imposed on them as a result of historical injustice and trauma. This scenario has repeatedly been highlighted by representatives of Black and Indigenous populations in the United States and Canada (Ball & O’Nell, 2016; Haskell & Randall, 2009; Proudfoot, 2023), and seems to also be relevant to the Sweden Finnish experience. Overall, many of the negative health behavior stereotypes described by our participants are shared by other minoritized ethnic and Indigenous groups of the Circumpolar North. For example, Greenlanders living in Denmark are typically depicted in Danish media as loud, drunk, and potentially violent, although a vast majority of Greenlanders in Denmark lead lives similar to that of any other Dane (Terpstra, 2015). Moreover, the harmful image of the ‘drunk male laborer’ is mirrored in the experiences of Irish labor migrants to England in the post-Second World War era (Hickman, 1998; Hickman & Ryan, 2020), as well as those of Polish labor migrants, both in England and Sweden (Fox et al., 2015; Runfors, 2021). It is, of course, fully possible to acknowledge and address health issues among Sweden Finns, such as alcohol-related mortality, and strive toward as more nuanced and less prejudiced public image of the Sweden Finnish population at the same time (more on that below). Potentially, the notion of *lateral violence*—a term originating in Indigenous contexts, used to describe the tendency among members of minoritized groups to direct frustration from everyday experiences of oppression toward other group members (Whyman et al., 2021)—could be useful for developing a more layered understanding of these social dynamics within the Sweden Finn community. Still, our participants clearly express a lack of helpful and available strategies in this regard. Several participants have themselves occasionally participated in stereotypical jokes about Sweden Finns in situations involving alcohol. In the moment, this might have been seen as a somewhat subversive self-mocking tactic; in the longer run, however, behaviors like these can contribute in internalizing negative self-images.

### War Trauma as a Transgenerational Phenomenon

Discussing the ambivalent topic of alcohol use among Sweden Finns, our participants stress the devastating and multilayered impact of the many wars that were waged on Finnish ground in the 20th century on the health and well-being of the population. The fact that this was raised by participants from the first, second, and third generations alike suggests that a substantial transgenerational impact persists. The collectively experienced war traumas have shaped contemporary Finland in multiple and complex ways; they might, for example, have created a certain harshness and pragmatism in terms of how Finns and Sweden Finns view work and social relationships, while also contributing to a sense of affinity and communal aspirations. This part of modern history is, apparently, relatively unknown in Sweden. Although the question of Swedish neutrality during the Second World War remains a subject of debate, Sweden was largely unharmed by the war, whereas war-torn Finland was plagued by destruction, poverty, and unemployment. The fundamentally different experiences during the Second World War and in the post-war era might have contributed to a certain cluelessness among the Swedish public that further alienated those Sweden Finns who were directly or indirectly affected by the wars.

The recognition of transgenerational aspects of war trauma is becoming more commonplace in Finland of today, in research as well as in public discourse. During and after the wars, soldiers, and civilians alike suffered various war-related traumas, such as the loss of loved ones, forced displacement, and the evacuation of so-called war children to neighboring countries. After the Second World War, the understanding of how to best support returning soldiers was rudimentary. A strong culture of silence meant that psychological trauma was rarely discussed openly. Rather than acknowledging that mental health problems could originate from traumatic war experiences, a common narrative included ideas about ‘weak mental constitution’ and ‘degeneration’ among sufferers, resulting in stigma and shame (Kivimäki, 2013). Thus, returning soldiers were left to deal with any psychological ailments on their own, without professional support; often, unresolved trauma led to destructive behaviors, such as alcohol abuse and domestic violence (Laurén & Malinen, 2021). Pervasive trauma and a societal culture of silence laid the ground for transgenerational transmission. A central notion in Finnish discourse on transgenerational trauma is *taakkasiirtymä* (Siltala, 2016), which can be translated as ‘transfer of burden.’ This concept was developed in the 1960s by psychiatrist and psychoanalyst Martti Siirala and echo modern understandings of the phenomenology of transgenerational trauma.

A similar post-war culture of silence has been documented in many other settings worldwide. For example, in the aftermath of the Cambodian genocide, it was noted that many people were simply ‘too poor to care’ about the past, i.e., they were preoccupied with their day-to-day survival and did not have the privilege of addressing the traumas they had suffered (Münyas, 2008). This observation appears to have some relevance for the Finnish and Sweden Finnish post-war experiences as well. In terms of grit and resilience, the oft-mentioned but somewhat elusive Finnish notion of sisu is described by our participants as both contributing to and emanating from a broader societal discourse on the importance of showing determination and carrying on in the face of adversity. Notably, the idea of sisu has also been an important element in the self-understanding of the Finnish diaspora (Taramaa, 2009). It may be tempting to think of sisu as an *idiom of resilience*—that is, a culturally sanctioned way of embodying, performing, and communicating adaptation and coping in the face of adversity (Kaiser & Weaver, 2019). Interestingly, however, the explicit and implicit moral components of this ‘typically Finnish’ steadfastness have also been linked to a rising tide of occupational burnout, as the historically and collectively rooted sisu encounters the individual-focused demands of the new economy (Funahashi, 2023). Our participants’ narratives share the same ambivalence: here, Finnish grit is a celebrated strength that, paradoxically, can also have deadly consequences. It is noteworthy that in Sweden, occupational burnout has—on somewhat similar moral grounds, though without the all-encompassing quality associated with Finnish sisu—been described as akin to a uniquely Swedish culture-bound syndrome (Friberg, 2006). This exceptionalist Swedish self-image is arguably flawed; indeed, the collective Finnish post-war experiences may have even further reinforced tendencies toward self-sacrifice compared to those seen in Sweden.

### The Importance of Cultural and Structural Competence and Sensitivity

Prejudice, stereotypes, and stigma also affect healthcare encounters. For example, our participants describe how older relatives are told by healthcare staff to be tough and to ‘stop whining,’ with reference to their Finnish background. Moreover, the image of Sweden Finns as generally reluctant to seek healthcare assistance is raised in most of our participant narratives. This notion appears to be somewhat of a double-edged sword. On the one hand, many Sweden Finns obviously tend to postpone help-seeking, for fear of being a burden or based on previous negative experiences. On the other hand, a stereotypical view of Sweden Finns as tough, stoic, and of few words might in itself become a barrier in healthcare contexts. Several participants describe how their symptom expressions have been neglected by majority-Swedish healthcare professionals, whereas healthcare professionals with a Finnish background have seen the very same symptom expressions as worthy of medical attention. Thus, it is not only a problem of avoidant behavior on the part of the patient, but also about healthcare providers’ lack of sensitivity toward cultural idioms of distress (Kaiser & Weaver, 2019; Nichter, 2010). This finding underscores the importance of cultural sensitivity, humility, and safety in healthcare (Danso, 2016; Kirmayer, 2012; Kleinman & Benson, 2006), as well as of ensuring that patients have the opportunity to describe sensitive issues such as health problems in the language that they are most comfortable with. Importantly, the relationship between language, thought, and expression goes deeper than what can be easily transmitted through literal translation (Imai et al., 2016), something that several of our participants have experienced.

In connection with this discussion about prejudice and stereotypes in healthcare, broader critical explorations of societal stigma become highly relevant. Here, stigma is understood not as a singular, interindividual phenomenon, but as a dynamic and multidimensional process embedded within social, cultural, and structural contexts (Pescosolido & Martin, 2015). Accordingly, understanding stigma and designing effective interventions require a systems-based approach—one that recognizes that stigma is rarely the sole source of disadvantage, but often intersects with various forms of structural inequality (Scambler, 2009). The need for an increased emphasis on structural competence in healthcare is therefore also evident, i.e., an ability to recognize how health is affected by broad social, political, and economic structures (Metzl & Hansen, 2014). This includes acknowledging and addressing prejudice and discrimination, rather than applying a routine ‘blindness’ regarding these issues. In order not to reproduce stereotypes, healthcare providers also need to be conscious about their own preconceived notions. For instance, in addressing substance use, it is important to be mindful of the prevailing stereotypical image of Sweden Finns as heavy drinkers while simultaneously not shying away from important discussions about a patient’s real alcohol-related health issues. There is no one-size-fits-all solution for how to best approach socially charged and potentially sensitive topics such as this, nor is it realistic for healthcare professionals to achieve in-depth knowledge about the sociocultural conditions affecting every minoritized group in society. Importantly, both cultural and structural competence should involve an emphasis on *becoming* rather than *being* competent (Cai, 2016)—they are not technical skills that you either have or do not have, but rather overarching attitudes that involve maintaining an active curiosity about the lived realities of one’s patients. Such curiosity, in turn, is helpful in creating ‘places for listening’ (Fassin, 2012) and establishing a platform from which to collaboratively address cultural and socioeconomic issues in healthcare.

Nevertheless, efforts to counteract stigma that rely solely on increasing knowledge or awareness are ultimately insufficient; social inclusion and structural reform are essential levers for lasting change (Pescosolido & Martin, 2015). Importantly, the need for community outreach initiatives that prioritize dialogue—enabling healthcare providers and policymakers to learn from underserved populations and more effectively address their needs—has been increasingly emphasized (Gilmore et al., 2023). This perspective can be understood as a response to paternalistic, ‘top-down’ public health strategies, which tend to frame unhealthy behaviors as matters of individual choice, often leading to victim blaming and stigmatization (Brewis & Wutich, 2019).

### Rigor and Trustworthiness

To the best of our knowledge, this is the first study with an explicit focus on experiences of health and healthcare among the general Sweden Finnish population. Most previous research on the health of people with a Finnish background in Sweden has relied on quantitative methods that do not allow for self-identification according to the principle that guides official minority legislation, i.e., participants have not been asked whether or not they actually see themselves as Sweden Finns. In the present study, in contrast, this topic has been thoroughly discussed, revealing a substantial ambivalence in relation to the officially recognized terminology. Another strength is the active involvement of a Sweden Finnish reference group in all phases of the study, ensuring that the research questions are relevant to the population as a whole and that the practical procedures are acceptable and inclusive.

This use of the officially recognized terminology, i.e., Sweden Finns, in recruitment might, however, also have limited our outreach. Our findings show that many participants have chosen to take part in the study in spite of an ambivalence toward this terminology, and it is likely that others have refrained from participating altogether for this very reason. As described in more detail in the methods section, we have made an effort to be clear about the many potential nuances of ethnic and cultural identity in our recruitment material. Nevertheless, we cannot readily get around the fact that inclusion in our target population ultimately depends on self-identification and that it is reasonable for those who do not see Sweden Finnishness as a relevant identity to not participate.

Since only three men chose to participate in the study, we cannot draw any far-reaching conclusions regarding gender differences. We can only speculate as to why we were not able to recruit more men. Gender norms might have contributed, in that women are perhaps generally more used to and comfortable with talking openly about sensitive and emotionally laden topics (Schwalbe & Wolkomir, 2001). To be clear, any lack of heterogeneity among participants need not be a major limitation, as our aim is not to produce universal or generalizable ‘truths’ but to explore how participants construct meaning within their specific contexts. Nevertheless, including more male participants could have offered a broader understanding of health and well-being among Sweden Finns.

Lastly, it can be noted that none of our participants came to Sweden as a so-called war child in 1939–1944. The former war children who are still alive are now in their eighties and nineties, which may have been an obstacle to participation. Furthermore, former war children might for various reasons not view themselves as belonging to the Sweden Finnish national minority to the same extent as other individuals with a Finnish background in Sweden. For readers interested in their experiences, there are a number of available qualitative studies that address this topic (Heilala, 2016; Lagnebro, 1994; Mattsson, 2018).

## Supplementary Information

Below is the link to the electronic supplementary material.

Supplementary file1 (DOCX 19 KB)

## Data Availability

The data that support the findings of this study are available in a fully anonymized and synthesized format from the corresponding author upon reasonable request.
