# Supplementary material for: What We Don't Speak of: Exploring the Impact of Historical Trauma and Discrimination on the Health and Well-Being of Sweden Finns
Source: Cult Med Psychiatry. 2026 Jan 19;50(1):5. doi: 10.1007/s11013-026-09969-0 (PMC12816104; doi:10.1007/s11013-026-09969-0)
Supplement: Supplementary file 1 — Supplementary file1 (DOCX 19 KB) [file 11013_2026_9969_MOESM1_ESM.docx]

**Supplementary Material: Interview Guide**

The questions in this semi-structured interview guide are intended to help the interviewer maintain a topical focus during the interview. They do not need to be asked verbatim or in a fixed order. Questions that are not relevant for a particular participant may be skipped, and the interviewer may also introduce supplementary questions not listed here, depending on the participant’s narrative.

**Identity and Background**

Do you identify as Sweden Finn? What other identities are important to you? Do you belong to any other national minority group?

What does your Sweden Finnish and/or Finnish background mean to you?

What does Finnishness mean to you?

In what ways do Finnish traditions and customs influence you? Do you miss any of them in Sweden? If so, how does that affect your health?

What significance does the Sweden Finnish community have for you? Have you participated in any Sweden Finnish associations? If so, do you think this has affected your well-being or health in any way?

What is your parents’ or your family’s connection to their Sweden Finnish and/or Finnish background?

How do you perceive the attitudes of the surrounding society toward you as a Sweden Finn, and how does this affect you? Please give examples from the media, social media, as well as your local community.

Do you perceive any prejudices or stereotypes about Sweden Finns? If so, how do you relate to them personally?

**General Health and Healthcare Encounters**

How would you describe your health today?

Do you think your current life situation affects your health in any way?

What helps you achieve or maintain a good quality of life?

What do you personally do to feel calm and safe when you are worried or troubled?

What factors do you consider essential for maintaining overall well-being, including physical, mental, social, and spiritual aspects?

When facing health problems, what strategies do you think you can use yourself to cope with them? Could you share any examples, including any related to mental health?

People often seek help for health problems outside the formal healthcare system, for instance through alternative or traditional medicine. Could you share any examples of this, including any related to mental health? These examples can concern yourself or someone else.

When it comes to your health, which parts of your background and sense of belonging do you feel are most important?

What does living in a metropolitan area mean to you as a Sweden Finn? Do you think it affects your health?

If you moved here from Finland, what does that experience mean to you, and do you feel it has had any impact on your health?

How can various public authorities help you and your family achieve or maintain a good quality of life? If needed, provide examples, such as childcare, school, elder care, income support, or unemployment assistance.

If you or your family sought support in a challenging life situation, how would you like that support to be provided? Do you think your background would influence the support you receive? How would you like your background and identity to be taken into account?

Have you ever been treated badly because of your background? If so, has it ever been by an official or authority figure? How has this affected you?

Have you ever experienced discrimination for any other reason, in Sweden or Finland?

Have you ever avoided contacting healthcare services because you were worried about being treated unfairly or poorly?

Has your family experienced mistreatment due to their background over time, and does this continue to affect you today?

If you or your family were facing health challenges, what kind of support would you like to have access to? Are there any barriers that might prevent you or your family from getting the help you need? Do you think your background could affect your ability to receive support?

Misunderstandings can sometimes occur between healthcare providers and patients due to differences in background or expectations. Could you share any examples of when this has happened? Did your background play a role? What are your views on healthcare providers’ knowledge of the Sweden Finnish national minority and their circumstances? Please describe any experiences you may have had. Have you ever felt mistreated in healthcare because of your Sweden Finnish identity?

What do you think could help improve interactions with healthcare services? How would you like healthcare—particularly mental health care—to be developed? What difference do you think it would make if healthcare providers had more knowledge about the Sweden Finnish community?

**Mental Health**

Who would you primarily turn to if you were experiencing mental health problems?

Would you describe any mental health problems differently to your family, friends, or others in your community than you would to healthcare providers?

Open question: Do you feel that certain mental health issues are more common among Sweden Finns?

If specific areas are not mentioned: Statistics indicate that suicide, suicide attempts, and alcohol-related problems are more common among Sweden Finns. What are your thoughts on this?

We know there are many reasons why people develop mental health problems. Based on your experiences, is there anything about being a Sweden Finn that affects mental health?

Are you aware of any mental health issues in your family? Have these topics been discussed? How would you advise a friend to seek help for mental health concerns in Stockholm? Do you think it makes a difference if the person is a Sweden Finn? Does language or other cultural factors matter? Is there anything you believe healthcare providers should consider when working with a Sweden Finn patient?

Is there anything else you would like to share? Are there any important topics I haven’t asked about?
